# A vector-based hypoplastic model for interface behavior of granular material

**DOI:** 10.1007/s11440-026-02995-7

**Published:** 2026-04-02

**Authors:** Xuan Kang, Ivan Zaboev, Hao Yang, Shun Wang, Wei Wu

**Affiliations:** 1 Institut für Geotechnik, Universität für Bodenkultur Wien, Feistmantelstrasse 4, A-1180 Vienna, Austria; 2 State Key Laboratory of Water Resources and Hydropower Engineering Science, Wuhan University, 299 Bayi Road, Wuhan, 430072 People’s Republic of China

**Keywords:** Granular materials, Hypoplastic model, Interface behavior, Shear-band analysis

## Abstract

This paper presents a vector-based hypoplastic model for the interface behavior of granular materials. By applying a unit normal vector, the original tensorial model is reduced to in-plane projections of stress and strain rate onto the contact surface. To model the shear-band formation in granular soils, a strain-gradient extension is incorporated into the vector-based hypoplastic model. By introducing an inherent length scale, a finite shear-band thickness in the post-localization regime can be reproduced. The proposed model inherits the simplicity of the original hypoplastic model while enabling shear-band analysis under interface conditions. Numerical simulations show that the proposed model captures the salient features of granular soil-structure interface behavior. In addition, the onset of localization and shear-band thickness are governed by the gradient boundary condition imposed at the contact surface.

## Introduction

The mechanical behavior of soil-structure interfaces has been widely studied due to its importance in geotechnical engineering applications, such as pile foundations [1, 21, 24], retaining structures [10, 29], and geosynthetic reinforcements [27]. The shear localization at the contact surface governs the majority of the mechanical response, which also depends strongly on the soil properties and interface characteristics. For granular materials, factors such as grain size distribution and particle crushability are recognized as key influences on the interface behavior [7, 13]. Numerous experimental approaches, including direct shear and simple shear tests [8, 25], have been employed to study the granular soil-structure interface behavior. However, numerical simulation of granular interface behavior remains challenging due to the lack of a reliable yet simple constitutive model that can be readily implemented in computational analyses. Consequently, the interface behavior remains one of the most challenging aspects of soil constitutive modeling.

Traditional constitutive descriptions of interface behavior have relied on extensions of plastic and elasto-plastic frameworks [4, 6, 14]. Based on yield surfaces and flow rules, elasto-plastic approaches have been widely adopted since they can capture strength mobilization and post-peak softening. Building on this foundation, various extended models have been proposed to address specific interface problems [18, 20, 23]. In recent decades, hypoplasticity has emerged as an alternative approach for modeling soil behavior using tensorial equations [46, 47]. Unlike classical plasticity, hypoplastic models are formulated without the concepts of yield surfaces, plastic potentials, or the decomposition of strain into elastic and plastic components. As a result, hypoplastic models are usually characterized by simple formulations and few parameters. Moreover, hypoplastic model possesses a single formulation for the incrementally nonlinear behavior without using a predefined criterion for loading and unloading. These features make hypoplastic models more straightforward and computationally efficient to implement compared with conventional elastoplastic formulations [11, 17, 37, 39–41, 48].

The first attempt to incorporate interface behavior into the hypoplastic framework was proposed by Gutjahr [16], where a one-dimensional hypoplastic interface model was developed based on the three-dimensional hypoplastic formulation with a predefined limit state surface [36]. This early model was capable of describing the influence of surface roughness on soil-structure interaction. On this basis, Arnold and Herle [3] extended the concept to two dimensions by formulating reduced stress and stretching tensors under 2D interface conditions. Afterwards, Stutz et al. [31, 33] further enhanced the model by incorporating in-plane stress effects for both granular and fine-grained soils. The numerical implementation of this extended model demonstrated the satisfactory performance of the hypoplastic framework for interface behavior within finite element analyses [32].

Except for interface behavior, shear-band formation in the post-localization regime also remains a longstanding challenge in constitutive modeling of granular materials. Such localization depends not only on boundary conditions and interface characteristics but also on the inherent length scale of the material itself [5, 34]. Conventional continuum theories, such as elastoplastic and hypoplastic models, lack this inherent length scale and therefore cannot capture the size-dependent behavior associated with shear-band formation. To overcome this limitation, a series of gradient-based models have been developed within existing constitutive frameworks to address practical problems like shear-band thickness [2, 50]. These higher-order models introduce a regularization mechanism that enables the localization problem to be resolved even at large strains.

This study follows the idea of Arnold and Herle [3] by simplifying the hypoplastic model into a vector form through the reduction of out-of-plane stress components. Building on the recently proposed Simhypo-sand model [43], the present approach describes interface behavior using the projections of the stress and strain-rate tensors onto the contact surface. For simplicity, these projected quantities are referred to as stress and strain-rate vectors. To evaluate the performance of the proposed model, numerical simulations under different loading conditions are carried out to compare with the original tensor model and the previous interface model. To conduct the shear-band analysis under interface conditions, a strain-gradient extension is introduced to the proposed model to introduce an inherent length scale. The influence of the contact surface on localization and shear-band thickness in granular soil is investigated by modifying the strain-gradient boundary condition at the interface.

## Vector-based hypoplastic model for interface behavior

### Hypoplastic framework

The general hypoplastic constitutive framework proposed by Wu and Kolymbas [44] can be written as:

1 $$\begin{aligned} \mathring{\boldsymbol{\sigma }}= \mathcal {L}(\boldsymbol{\sigma } ):\dot{\boldsymbol{\epsilon }} + {\textbf {N}}(\boldsymbol{\sigma })\left\| \dot{\boldsymbol{\epsilon }} \right\| \end{aligned}$$

where $\boldsymbol{\sigma }$ is the Cauchy stress tensor; $\dot{\boldsymbol{\epsilon }}$ is the strain rate (stretching) tensor. The tensorial functions $\mathcal {L}$ and ${\textbf {N}}$ are of the fourth and second order, respectively. The colon ${\textbf {:}}$ denotes an inner product between two tensors. $\left\| \dot{\boldsymbol{\epsilon }} \right\|$ stands for the Euclidean norm of the stretching tensor. The Jaumann stress rate tensor $\mathring{\boldsymbol{\sigma }}$ is defined in terms of the material time-derivative of the Cauchy stress tensor $\boldsymbol{\sigma }$ and the spin tensor $\dot{\boldsymbol{\omega }}$:

2 $$\begin{aligned} \mathring{\boldsymbol{\sigma }} = \dot{\boldsymbol{\sigma }} + \boldsymbol{\sigma } \dot{\boldsymbol{\omega }} - \dot{\boldsymbol{\omega }} \boldsymbol{\sigma } \end{aligned}$$

The above stretching and spin tensors are related to the velocity gradient tensor through3where $\boldsymbol{v}$ is the velocity and $\triangledown$ is the gradient operator.

Based on the basic model proposed by Wu et al. [47], Wang et al. [43] proposed the Simhypo-sand model for describing the behavior of granular materials, given as:

4 $$\begin{aligned} \mathring{\boldsymbol{\sigma }} = f_s\Big [ \textrm{tr}(\boldsymbol{\sigma })\dot{\boldsymbol{\epsilon }} + f_v \textrm{tr}(\dot{\boldsymbol{\epsilon }})\boldsymbol{\sigma } + a^2 \frac{\textrm{tr}(\boldsymbol{\sigma }\dot{\boldsymbol{\epsilon }})}{\textrm{tr}(\boldsymbol{\sigma })}\boldsymbol{\sigma } + f_da (\boldsymbol{\sigma }+\boldsymbol{\sigma }^{*}) \left\| \dot{\boldsymbol{\epsilon }} \right\| \Big ] \end{aligned}$$

where $\boldsymbol{\sigma }^{*}$ is the deviatoric stress tensor, defined by $\boldsymbol{\sigma }^{*}= \boldsymbol{\sigma } - 1/3\textrm{tr}(\boldsymbol{\sigma })\boldsymbol{\textrm{I}}$ with $\boldsymbol{\textrm{I}}$ being the second-order unit tensor. The multipliers $f_s$ and $f_v$ account for the stiffness and volumetric response of the granular materials, respectively. The multiplier $f_d$ is the density factor. The parameter *a* a material constant controlling the failure surface. The Simhypo-sand model requires only seven parameters to reproduce the key features of granular material behavior and has been successfully applied in large-deformation numerical simulations [19, 42, 43, 51]. In this study, this tensorial model is reduced to a vector formulation for the interface behavior of granular materials. Therefore, all the multipliers retain the same physical meanings and are subsequently adopted in the vector-based hypoplastic model, where the detailed are given in Sect. 2.4.

### In-plane stress condition

**Fig. 1:**
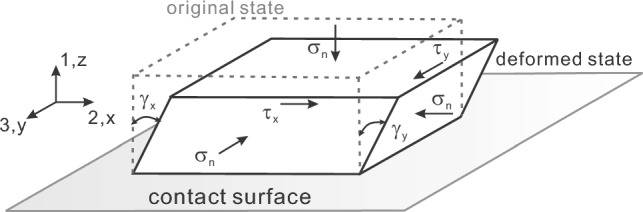
Stress and strain components acting on the soil-structure interface, illustrated with the local interface-aligned coordinate system and the global reference system

To consider in-plane stress condition for interface behavior, we inherit the assumption proposed by Arnold and Herle [3], which is illustrated in Fig. 1. The interface behavior is treated as a two-dimensional mechanical problem, while stresses and strain rates are expressed in three-dimensional tensor form to enable a consistent evaluation of constitutive invariants. For the local interface-aligned coordinate system, the stress tensor $\boldsymbol{\sigma }$ has the stresses $\sigma _{11} = \sigma _n$, $\sigma _{12} = \tau _{x}$ and $\sigma _{13} = \tau _{y}$, in which $\sigma _n$ is the normal contact stress (compression negative) and $\tau _{x}$ as well as $\tau _{y}$ are the shear stresses in the 2D contact plane. The remaining normal stresses are introduced as auxiliary quantities and are taken as $\sigma _{22} = \sigma _{33} = \sigma _{n}$, and the out-of-plane shear stress is assumed $\sigma _{23} = 0$.

Thus, the stress tensor reduces to:

5 $$\begin{aligned} \boldsymbol{\sigma }^{\text {full}} = \begin{bmatrix} \sigma _{11} &  \sigma _{12} &  \sigma _{13} \\ \sigma _{12} &  \sigma _{22} &  \sigma _{23} \\ \sigma _{13} &  \sigma _{23} &  \sigma _{33} \end{bmatrix} \Rightarrow \boldsymbol{\sigma }^{\text {reduce}}= \begin{bmatrix} \sigma _n &  \tau _{x} &  \tau _{y} \\ \tau _{x} &  \sigma _n &  0 \\ \tau _{y} &  0 &  \sigma _n \end{bmatrix} \end{aligned}$$

where $\boldsymbol{\sigma }^{\text {full}}$ denotes the full stress tensor and $\boldsymbol{\sigma }^{\text {reduce}}$ denotes the reduced stress tensor under in-plane stress condition.

The strain rate tensor is described analogously, that strain rate components are reduced to represent a simplified interface condition. The strain rates $\dot{\varepsilon }_{ii}$ with $i \in \{1,2,3\}$ are set to be equal to the strain rate $\dot{\varepsilon }_{n}$ perpendicular to the contact. The shear strain rates $\dot{\varepsilon }_{12}$ and $\dot{\varepsilon }_{13}$ are obtained from $\dot{\gamma }_{x}/2$ and $\dot{\gamma }_{y}/2$. Because of Eq. (5), $\dot{\varepsilon }_{23} = 0$ is required. This leads to the strain rate tensor as:

6 $$\begin{aligned} \dot{\boldsymbol{\epsilon }}^{\text {full}} = \begin{bmatrix} \dot{\varepsilon }_{11} &  \dot{\varepsilon }_{12} &  \dot{\varepsilon }_{13} \\ \dot{\varepsilon }_{12} &  \dot{\varepsilon }_{22} &  \dot{\varepsilon }_{23} \\ \dot{\varepsilon }_{13} &  \dot{\varepsilon }_{23} &  \dot{\varepsilon }_{33} \end{bmatrix} \Rightarrow \dot{\boldsymbol{\epsilon }}^{\text {reduce}} = \begin{bmatrix} \dot{\varepsilon }_{n} &  \tfrac{\dot{\gamma }_{x}}{2} &  \tfrac{\dot{\gamma }_{y}}{2} \\ \tfrac{\dot{\gamma }_{x}}{2} &  \dot{\varepsilon }_{n} &  0 \\ \tfrac{\dot{\gamma }_{y}}{2} &  0 &  \dot{\varepsilon }_{n} \end{bmatrix} \end{aligned}$$

where $\dot{\boldsymbol{\epsilon }}^{\text {full}}$ denotes the full strain rate tensor and $\dot{\boldsymbol{\epsilon }}^{\text {reduce}}$ denotes the reduced strain rate tensor under in-plane stress condition.

### Simplified vector notation

In the following, a unit normal vector $\boldsymbol{n}$ is introduced to further simplify the reduced stress and strain rate tensor under in-plane stress conditions:

7 $$\begin{aligned} \boldsymbol{n}=\begin{bmatrix} 1\\ 0\\ 0 \end{bmatrix} \end{aligned}$$

where it depends on the orientation of the interface. For a general contact surface, the unit normal vector is defined according to the interface orientation, and the same projection procedure is applied consistently. Here, we simply choose $\boldsymbol{n} = [1,0,0]^\text {T}$ for illustration, without loss of generality.

Therefore, the interface stresses and strain rates can be reduced to their projections onto the contact plane. Hereafter, we call these projected quantities stress and strain rate vectors. The stress vector ($\boldsymbol{\sigma }_{{\textbf {v}}}$) is obtained as:

8 $$\begin{aligned} \boldsymbol{\sigma }_{{\textbf {v}}} = \begin{bmatrix} \sigma _n &  \tau _{x} &  \tau _{y} \\ \tau _{x} &  \sigma _n &  0 \\ \tau _{y} &  0 &  \sigma _n \end{bmatrix} \cdot \begin{bmatrix} 1\\ 0\\ 0 \end{bmatrix} = \begin{bmatrix} \sigma _{n} \\ \tau _x \\ \tau _y \end{bmatrix} \end{aligned}$$

and strain rate vector ($\dot{\boldsymbol{\epsilon }}_{{\textbf {v}}}$) is obtained as:

9 $$\begin{aligned} \dot{\boldsymbol{\epsilon }}_{{\textbf {v}}} = \begin{bmatrix} \dot{\varepsilon } &  \tfrac{\dot{\gamma }_{x}}{2} &  \tfrac{\dot{\gamma }_{y}}{2} \\ \tfrac{\dot{\gamma }_{x}}{2} &  \dot{\varepsilon } &  0 \\ \tfrac{\dot{\gamma }_{y}}{2} &  0 &  \dot{\varepsilon } \end{bmatrix} \cdot \begin{bmatrix} 1\\ 0\\ 0 \end{bmatrix} = \begin{bmatrix} \dot{\epsilon } \\ \frac{\dot{\gamma }_{x}}{2} \\ \frac{\dot{\gamma }_{y}}{2} \end{bmatrix} \end{aligned}$$

Following Eq. (4), the vector-based hypoplastic model for interface behavior is given as:

10 $$\begin{aligned} \dot{\boldsymbol{\sigma }}_{{\textbf {v}}}= f_s\Big [\sigma _n{\dot{\boldsymbol{\epsilon }}_{{\textbf {v}}}} + f_v\dot{\epsilon }_{n}\boldsymbol{\sigma }_{{\textbf {v}}} + a^2\frac{\boldsymbol{\sigma }_{{\textbf {v}}}\cdot \dot{\boldsymbol{\epsilon }}_{{\textbf {v}}}}{\sigma _n}\boldsymbol{\sigma }_{{\textbf {v}}} + f_da(\boldsymbol{\sigma }_{{\textbf {v}}}+\boldsymbol{\sigma }_{{\textbf {v}}}^*)\left\| \dot{\boldsymbol{\epsilon }}_{{\textbf {v}}}\right\| \Big ] \end{aligned}$$

where $\sigma _n$ and $\dot{\epsilon }_n$ representing the stress and strain rate component perpendicular to the interface, obtained by:

11 $$\begin{aligned} \boldsymbol{\sigma }_{\boldsymbol{n}} = \boldsymbol{\sigma }_{{\textbf {v}}} \cdot \boldsymbol{n}, \quad \dot{\epsilon }_{n} = \dot{\boldsymbol{\epsilon }}_{{\textbf {v}}} \cdot \boldsymbol{n} \end{aligned}$$

The deviatoric stress is derived by:

12 $$\begin{aligned} \boldsymbol{\sigma }_{{\textbf {v}}}^* = \boldsymbol{\sigma }_{{\textbf {v}}} - (\boldsymbol{\sigma }_{{\textbf {v}}} \cdot \boldsymbol{n})\boldsymbol{n} \end{aligned}$$

The vector formulation relies on scalar products of stress and strain rate vectors, which remain invariant under coordinate rotations. As a result, the proposed model can be applied in any coordinate system, allowing interfaces of arbitrary orientation, e.g., inclined, vertical, or horizontal, to be treated without additional transformations. In earlier hypoplastic interface models [3, 33], tensor components were arranged in a vector-like format, but they remained tensorial quantities and therefore required rotation according to tensor transformation rules. In contrast, the present formulation uses true vectors obtained from projecting stress and strain rate onto the interface plane. These quantities transform as ordinary vectors and can be rotated using standard geometric rules, without relying on tensor-specific notation.

### Model parameter

Since the vector-based model constitutes a dimensional reduction of the tensorial hypoplastic law, the original multipliers of the tensor model (Eq. 4) cannot be directly used. In order to achieve equivalence in numerical predictions, the parameters were rescaled such that the stress–strain responses of the vector and tensor formulations coincide. For the vector model, the reduced dimensionality of stress and strain space results in the vanishing factor of the denominator for the stiffness factor. The multiplier $f_s$ accounts for the stiffness response is given as:

13 $$\begin{aligned} f_s= -\frac{E_i}{(1+\nu _i)\sigma _c} \end{aligned}$$

which ensures that both formulations produce consistent initial stiffness under normal loading. $E_i$ and $\nu _i$ are the material parameters representing for initial shear modulus and the initial Poisson’s ratio, respectively. It is noted that all material parameters used in this study are scalar model parameters. Therefore, no anisotropy between in-plane directions is implied. $\sigma _c =100$ kPa is used for material calibration.

The multiplier $f_v$ accounts for the volumetric response becomes:

14 $$\begin{aligned} f_v = \frac{3\nu _i+a\sqrt{1+2\nu _i^2}}{2(1-2\nu _i)}- \frac{5a^2}{6} \end{aligned}$$

in which *a* is related to the critical state value of the normalized deviatoric stress $\Vert \boldsymbol{\sigma }_c^* \Vert /\textrm{tr}(\boldsymbol{\sigma }_c)$. This parameter is modified in the vector formulation because the stress state is represented directly in terms of normal and shear components, rather than through tensorial invariants:

15 $$\begin{aligned} a = \frac{\sqrt{3}(2\sqrt{2} - \sin \phi _c)}{4\sqrt{2} \sin \phi _c} \end{aligned}$$

in which $\phi _c$ is the critical state friction angle. This adjustment ensures that the resulting vector model reproduces the same failure envelope as the tensorial model under equivalent loading conditions. Due to the reduced vector formulation, some multipliers inherited from the original tensor-based model require mathematical modifications to ensure equivalence. Accordingly, the physical meanings and constitutive roles of the multipliers remain unchanged.

The multiplier $f_d$ is a density factor, incorporates the critical state of granular materials, it remain unchanged in the vector model as:

16 $$\begin{aligned} f_{d} = \left( \frac{e - e_{d}}{e_{c} - e_{d}} \right) ^{\alpha } \end{aligned}$$

where *e*, $e_{d}$ and $e_{c}$ are the current, minimum, and critical state void ratios; $\alpha$ is a constant that controls the degree of strain softening [17, 37, 38]. The logarithmic critical state line proposed by Li and Wang [22] is adopted. The function of critical state void ratio $e_{c}$ dependent on the stress level is adopted as:

17 $$\begin{aligned} e_{c} = e_{\Gamma } \exp \left[ -\zeta \left( \frac{p}{p_{a}} \right) ^{\xi } \right] \end{aligned}$$

where $e_{\Gamma }$, $\zeta$, and $\xi$ are material parameters for characterizing the critical state of granular materials. $p'$ is the effective mean stress and $p_a$ is the atmospheric pressure for normalization.

The evaluation of void ratios at current and critical states give rise to different values of $f_d$, which is less than 1 for a dense state, greater than 1 for a loose state, and equals 1 at the critical state [43]. Here, the critical state refers to the critical-state behavior of the granular material. Clearly, the size of the yield surface evolutes with the variation of the density function $f_d$ before the material reaching the critical state ($f_d=1$ at the critical state). This suggests that the threshold yield stress for starting flow is also related to the initial condition, i.e., void ratio, of the granular material. These parameters depend only on critical state mechanics and are not affected by the dimensional reduction of the constitutive law.

The vector-based hypoplastic model contains seven parameters: $E_i$, $v_i$, $\phi _c$, and $\alpha$ for the hypoplastic model, $e_\Gamma$, $\lambda$, and $\xi$ for the critical state of granular materials. Specifically, $E_i$ (initial elastic modulus), $\nu _i$ (initial Poisson’s ratio), and $\phi _c$ (critical state friction angle) can be obtained from a single triaxial compression test with confining pressure of 100 kPa. $\alpha$ can be determined by trial and error based on the density effect on strain softening or hardening. The critical state parameters $e_{\Gamma }$, $\zeta$, and $\xi$ can be measured from an $e-\text {log}(p')$ plot of critical states based on three triaxial compression tests. Detailed information about all the material parameter can be referred to [11, 12, 43].

## Higher-order extensions for shear-band analysis

### Gradient model

Existing hypoplastic interface models have been well-developed to describe the mechanical response of granular materials at interfaces. However, they are not able to simulate shear-band formation or capture localization in the post-localization regime. For advanced numerical applications, it is important to recognize that granular materials display scale-dependent behavior, which cannot be fully addressed within conventional continuum theories lacking inherent length scales [5, 34]. To overcome this limitation, a strain-gradient extension of the proposed vector-based hypoplastic model is introduced. By incorporating a higher order gradient of the strain rate into the constitutive formulation, the model is able to regularize the post-localization response and predict a finite shear-band thickness near the interface.

The framework given by Eq. (1) shows hypoplastic model contains a linear and nonlinear tensorial function. The strain-gradient extension adopted here follows Osinov and Wu [26] and is introduced to regularize strain localization under in-plane stress conditions. In the present study, a parameter $\lambda$ accounting for the inherent length scale is introduced in the linear function by replacing strain rate $\dot{\boldsymbol{\epsilon }}$ with $\dot{\boldsymbol{\epsilon }} - \lambda ^2\nabla ^2\dot{\boldsymbol{\epsilon }}$, where $\nabla \dot{\boldsymbol{\epsilon }}$ is the Laplacian of the strain rate and $\lambda$ is assumed to depend on the mean grain diameter of the granular material. In certain boundary value problems, such as triaxial compression, the spin-related terms ($\boldsymbol{\sigma } \dot{\boldsymbol{\omega }} - \dot{\boldsymbol{\omega }} \boldsymbol{\sigma }$) in the Jaumann rate (Eq. 2) can be neglected, and the stress rate reduces effectively to the material derivative. However, for problems involving significant stress-axis rotations, such as interface shearing, these terms must be updated with an extended stress rate to ensure objectivity. While the kinematic relations of the velocity field $\boldsymbol{v}$ in Eq. (3) remain unchanged, the strain-gradient extension is introduced through a regularized strain rate and a consistently modified stress rate. The Laplacian originates from a spatial Taylor expansion of the field quantity, followed by averaging over a sphere of small radius *R* and neglecting higher-order terms $o(R^2)$. Therefore, Eq. (2) becomes:

18 $$\begin{aligned} \overset{\triangle }{\boldsymbol{\sigma }} = \dot{\boldsymbol{\sigma }} + \boldsymbol{\sigma } (\dot{\boldsymbol{\omega }} - \lambda ^2\nabla ^2\dot{\boldsymbol{\omega }}) - (\dot{\boldsymbol{\omega }} - \lambda ^2\nabla ^2\dot{\boldsymbol{\omega }}) \boldsymbol{\sigma } \end{aligned}$$

where $\nabla ^2\dot{\boldsymbol{\omega }}$ is the Laplacian of $\dot{\boldsymbol{\omega }}$, and $\lambda$ is the length-scale parameter. While the spin tensor $\dot{\boldsymbol{\omega }}$ itself is not objective, its Laplacian $\nabla ^2\dot{\boldsymbol{\omega }}$ is frame-indifferent. To see this, consider two Cartesian systems that coincide but rotate relative to each other. The corresponding velocity fields differ by a rigid-body rotation, producing an antisymmetric spin increment $\dot{\boldsymbol{\omega }}_0$ independent of spatial position. Since $\dot{\boldsymbol{\omega }}_0$ is constant, its derivatives vanish, and the spatial derivatives of $\dot{\boldsymbol{\omega }}$ remain identical in both frames. Detailed information can be found in Osinov and Wu [26].

With the use of the extended stress rate (Eq. 18), the proposed strain-gradient extension of Eq. (1) becomes

19 $$\begin{aligned} \overset{\triangle }{\boldsymbol{\sigma }}= \mathcal {L}(\boldsymbol{\sigma } ):(\dot{\boldsymbol{\epsilon }} - \lambda ^2\nabla ^2 \dot{\boldsymbol{\epsilon }} ) + {\textbf {N}}(\boldsymbol{\sigma })\left\| \dot{\boldsymbol{\epsilon }} \right\| \end{aligned}$$

### Inherent length scale

The approach used in elasto-plastic models [34, 35] is adopted here to study the inherent-length analysis for the hypoplastic model. We assume sinusoidal, spatially periodic velocity fields that satisfy equilibrium without violating the gradient hypoplastic law. Let the velocity depend on $x_1$ only having $v_i(x_1) = a_i \sin (k x_1)$ with wavelength $l = {2\pi }/{k}$. Imposing constant stress of $\dot{\sigma }_{1i} = 0$ on the extended model (Eq. 19) leads to the following governing ordinary differential equation for each component:

20 $$\begin{aligned} \eta _{ij} \frac{d v_j}{d x_1} - \lambda ^2 \eta _{ij} \frac{d^3v_j}{d x_1^3} + b_i ||\dot{\boldsymbol{\epsilon }}|| = 0, \quad i = 1, 2, 3 \end{aligned}$$

where $\eta _{ij}$ and $b_i$ are functions of the stress components and the void ratio, respectively. For $v_i(x_1) = a_i \sin (k x_1)$,

21 $$\begin{aligned} \Vert \dot{\boldsymbol{\epsilon }}|| = k s \cos (k x_1) \sqrt{a_1^2 + \frac{1}{2} a_2^2 + \frac{1}{2} a_3^2}, \quad s= \text {sgn} (\text {cos}(k x_1)) \end{aligned}$$

Collecting the $\cos (k x_1)$ terms gives a nonlinear algebraic system for the normalized amplitudes $\alpha _i= a_i/ \sqrt{a_1^2 + \frac{1}{2} a_2^2 + \frac{1}{2} a_3^2}$, which admits non-trivial solutions only if a stress-dependent condition holds. Introducing determinants $\Lambda _0$, $\Lambda _1$, $\Lambda _2$, $\Lambda _3$, which constitutes the amplitudes $\alpha _i$ and is the function of $\eta _{ij}$ and $b_i$ (see Appendix B), the equation can be obtained as:

22 $$\begin{aligned} (1 + \lambda ^2 k^2)^2 = \frac{\Lambda _1^2 + \frac{1}{2} \Lambda _2^2 + \frac{1}{2} \Lambda _3^2}{\Lambda _0^2} \end{aligned}$$

and therefore the normalized inherent length is achieved as:

23 $$\begin{aligned} \frac{l}{\lambda } = 2\pi \left( \sqrt{\frac{\Lambda _1^2 + \frac{1}{2} \Lambda _2^2 + \frac{1}{2} \Lambda _3^2}{\Lambda _0^2}} - 1 \right) ^{-1/2} \end{aligned}$$

### Shear-band analysis for the non-gradient equation

For the non-gradient hypoplastic model, shear-band formation can be analyzed by considering a velocity field that is continuous but undergoes a jump in its gradient across a plane $x_1 = const$. The condition for shear-band initiation is expressed as

24 $$\begin{aligned} \llbracket \dot{\sigma }_{1i} \rrbracket = 0, \quad i = 1, 2, 3 \end{aligned}$$

leading to the system

25 $$\begin{aligned} \eta _{ij} \llbracket \frac{d v_j}{d x_1} \rrbracket + b_i \llbracket \Vert \dot{\boldsymbol{\epsilon }} \Vert \rrbracket = 0, \quad i = 1, 2, 3 \end{aligned}$$

where $\eta _{ij}$ and $b_i$ are the same coefficients as in the gradient solution (Eq. 20). The solution gives

26 $$\begin{aligned} \llbracket \frac{d v_j}{d x_1} \rrbracket = \llbracket \Vert \dot{\boldsymbol{\epsilon }} \Vert \rrbracket \frac{\Lambda _j}{\Lambda _0}, \quad j = 1, 2, 3 \end{aligned}$$

with determinants $\Lambda _0$, $\Lambda _1$ defined as before.

To ensure physical admissibility, the jumps must satisfy the inequality

27 $$\begin{aligned} \Vert \llbracket \dot{\boldsymbol{\epsilon }} \rrbracket \Vert ^2 \ge \left( \Vert \dot{\boldsymbol{\epsilon }}^+ \Vert - \Vert \dot{\boldsymbol{\epsilon }}^- \Vert \right) ^2 \end{aligned}$$

which reduces to the condition

28 $$\begin{aligned} \llbracket \frac{d v_1}{d x_1} \rrbracket ^2 + \frac{1}{2} \llbracket \frac{d v_2}{d x_1} \rrbracket ^2 + \frac{1}{2} \llbracket \frac{d v_3}{d x_1} \rrbracket ^2 \ge \llbracket \Vert \dot{\boldsymbol{\epsilon }} \Vert \rrbracket ^2 \end{aligned}$$

Substituting Eq. (26) into Eq. (28), we obtain

29 $$\begin{aligned} \Lambda _1^2 + \frac{1}{2}\Lambda _2^2 + \frac{1}{2}\Lambda _3^2 \ge \Lambda _0^2 \end{aligned}$$

In summary, the shear-band criterion derived from the non-gradient formulation leads to the inequality in Eq. (29), which is equivalent to the existence condition for periodic velocity fields. This establishes a consistent link between bifurcation analysis and the potential onset of shear localization in the classical hypoplastic framework.

## Model validation and applications

### Model evaluations

To evaluate the performance of the proposed vector-based hypoplastic model under in-plane stress conditions, numerical simulations were conducted and compared with results from the Simhypo-Sand model (referred to tensor model) for granular materials. Three types of boundary conditions were considered, i.e., constant normal load (CNL), constant volume (CV), and constant normal stiffness (CNS), which are commonly adopted in studies of soil-structure interface behavior [9]. The material parameters used in the simulations are given in Table 1, and two initial void ratios ($e_0 = 0.83$ and $e_0 = 0.94$) were employed to represent different initial density states. Note that the simulations in this section are intended as benchmark tests for comparing the preformances of both vector-based and tensor-based models under interface boundary conditions.

**Table 1:** Parameters for simple shear tests of granular material

Parameter	$E_i$ (MPa)	$\phi _c$ ($^\circ$)	$\nu _i$	$e_d$	$e_{\Gamma }$	$\zeta$	$\xi$	$\alpha$
Value	10.0	25	0.28	0.55	0.95	0.102	0.807	0.5

**Fig. 2:**
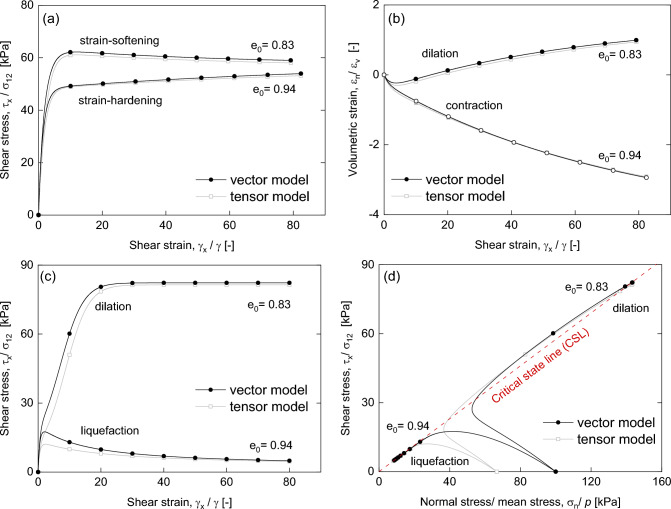
Model predictions of vector model (Eq. 10) and tensor model (Eq. 4) for the interface behavior of granular materials with different initial void ratios under conditions of (**a**, **b**) constant normal load (CN) and (**c**, **d**) constant volume conditions (CV)

The constant normal load (CNL) condition is defined by $K = 0$, $\dot{\boldsymbol{\sigma }} = 0$, and $\dot{\varepsilon } \ne 0$, where the normal stiffness is expressed as $K = \dot{\sigma } / {\dot{\varepsilon }}$. This boundary condition is the most commonly adopted in interface shear tests performed with direct shear devices. The simulation results of the CNL tests with different initial void ratios are shown in Figs. 2a–b as the stress–strain response and volumetric change, respectively. Due to the influence of initial density, the sand samples exhibit distinct stress–strain trends but ultimately converge toward an approximation at the critical state. The dense sample ($e_0$ = 0.83) give rise to strain-softening behaviours, whereas the loose sample ($e_0$ = 0.94) shows strain-hardening behaviour in shear stress. Correspondingly, dilative and contractive deformation are observed for the dense and loose states, respectively. Under the in-plane stress condition of the interface test, the vector-based model provides predictions closely matching those of the tensor model, confirming the consistency and reliability of the parameter calibration.

The constant volume (CV) condition is defined by $K = \infty$, $\dot{\boldsymbol{\sigma }} \ne 0$, and $\dot{\varepsilon } = 0$. The constant volume (CV) and the aforementioned constant normal load (CNL) are referred as the upper and lower limits in interface testing. In specific, the CNL condition corresponds to zero normal stiffness ($K = 0$), allowing free normal deformation under constant stress, whereas the CV condition corresponds to infinite stiffness ($K = \infty$), preventing any normal deformation. The numerical simulations of CV tests are shown in Fig. 2c-d. Under CV conditions, two distinct shearing responses are observed, which are commonly reported by undrained laboratory tests on sands with different initial densities [49]. Sand samples exhibit dilation under isochoric shearing with an initial dense state, leading to shear hardening until the critical state is reached. Although slight differences in peak strength are caused by the reduced stress representation in the vector model, the critical-state behavior remains consistent. In contrast, the loose sample undergoes a pronounced reduction in shear strength, eventually approaching a constant residual strength or complete liquefaction at the critical state.

Although the vector model reproduces a similar response of the shear stress compared to the tensor model, the stress path show in Fig. 2d shows the difference in initial state. This can be explained by that in the reduced vector formulation, only the stresses acting on the interface surface enter the model. As a consequence, an initially oedometric state is interpreted in the same way as isotropic stress state, leading to a slight overestimation of the liquefaction response. Despite this difference in the initial mean stress definition, the condition represents the same physical situation of a normal compression $\sigma _n$ acting across the interface as assumed in the vector-based model. This difference mainly affects the initial response of the undrained stress path, while the predicted critical-state behavior remains unchanged.

**Fig. 3:**
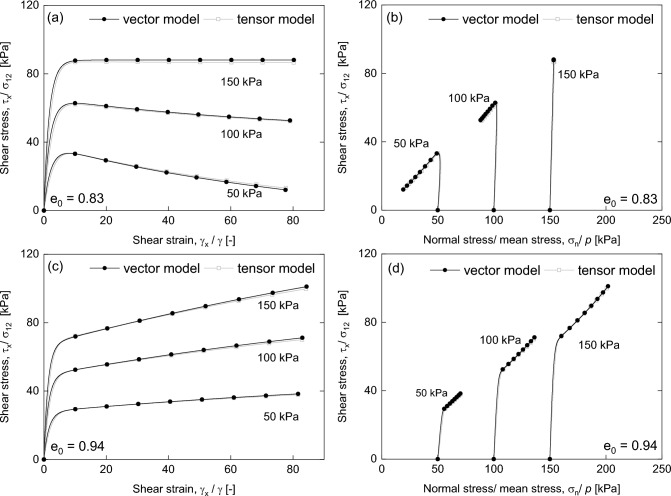
Model predictions of vector model (Eq. 10) and tensor model (Eq. 4) for the interface behavior of granular materials under constant normal stiffness with (**a**, **b**) initial dense state ($e_0 = 0.83$) (**c**, **d**) initial loose state ($e_0 = 0.94$)

The constant normal stiffness (CNS) condition is defined by *K* = constant, $\dot{\boldsymbol{\sigma }} \ne 0$, and $\dot{\varepsilon } \ne 0$. Different with CNL and CV conditions, CNS lies between these two extremes and represents a more realistic boundary constraint in soil–structure interaction problems. The results from the CNS comparisons are presented in Fig. 3. The applied constant normal stiffness is 1000 kPa, and the normal stress varies from 50 to 150 kPa. Both the vector and tensor formulations predict similar shear stress–strain responses, exhibiting consistent trends across all confining levels. For the dense state ($e_0 = 0.83$), a general strain-softening behavior is observed and becomes more pronounced as the small normal stress levels (Fig. 3a). The corresponding stress paths in Fig. 3b indicate a progressive approach toward the critical state line, representing a typical liquefaction-like response. At the highest normal stress of 150 kPa, the stress path shows a smoother transition and a mild post-peak softening trend.

For the samples with initial loose state (Fig. 3c), the shear stress–strain responses exhibit a typical strain-hardening behavior without post-peak, indicating a typical behavior characteristic of loose sand. The stress paths shown in Fig. 3d reveal that in the $\sigma _{n}$–$\tau _{x}$ plane, the sand yield a monotonic increase in both shear and normal stresses, showing a contractive response affected by loose state under drained loading. The numerical predictions of three boundary conditions suggest that the vector model is capable of capturing the salient features of granular materials, such as dilatancy and static liquefaction during interface tests. Additionally, the close agreement between the vector model and the original tensor model highlights the robustness of the vector reduction and its consistency within the hypoplastic framework.

**Fig. 4:**
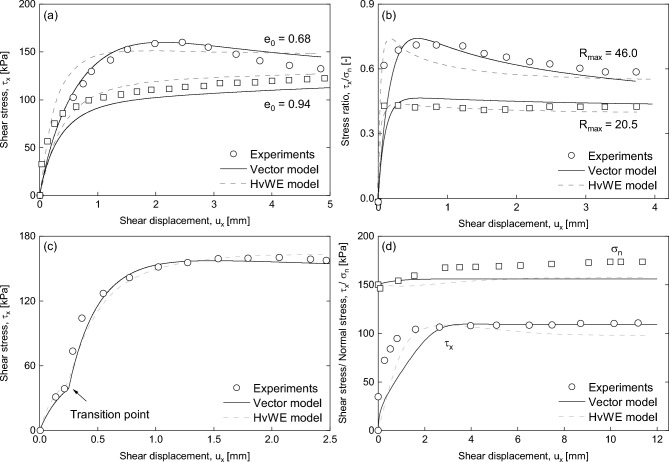
Comparison of experimental data from literature and hypoplastic models (vector model and HvWE model [31]). **a** CNL tests using Hostun sand under normal stress of 300 kPa with rough interface [30]. **b** CNL tests using Toyura sand under normal stress of 78 kPa with steel interface with different roughness [31]. **c** CNL tests using Density sand under staged normal stresses from 102 kPa to 274 kPa [15]. **d** CNS tests using Ticino sand under normal stress of 150 kPa with *K* = 100 kPa [28]

### Model validation with experimental data

To further evaluate the predictive capability of the proposed vector model, a series of direct shear test simulations were performed and compared with experimental results reported in the literature. In addition, the model performance was compared with the HvWE model developed by Stutz et al. [31], which is based on the hypoplastic framework of Von Wolffersdroff [36]. The simulation results are presented in Fig. 4, and the parameters used in the analyses are listed in Table 2.

The experimental data in Fig. 4a correspond to constant normal load (CNL) interface shear tests on Hostun sand conducted by Shahrour and Rezaie [30]. The tests were carried out under a constant normal stress of 300 kPa and a total shear displacement of 5 mm, with two initial void ratios representing dense and loose states, i.e., $e_0 = 0.68$ and $e_0 = 0.94$, respectively. The interface used in the experiments was defined as rough. Both models gives satisfied predictions, which matches with the experimental observations. Compared to the HvWE model, the vector model accurately reproduces the interface responses of sands with different initial densities. In particular, the strain-softening behavior of dense sand and the strain-hardening response of loose sand are well captured, demonstrating the improved predictive performance over the HvWE model on granular materials.

**Table 2:** Parameters for direct shear tests of sand-structure interfaces

	$E_i$ (MPa)	$\phi _c$ ($^\circ$)	$\nu _i$	$e_d$	$e_{\Gamma }$	$\zeta$	$\xi$	$\alpha$
Hostun sand	2.6	19	0.13	0.48	0.96	0.14	0.36	0.4
Toyura sand	13.0	19	0.13	0.45	0.96	0.14	0.36	0.4
Density sand	7.0	20	0.33	0.45	0.98	0.12	0.22	0.35
Ticino sand	10.0	30	0.15	0.48	0.85	0.502	0.08	0.4

The test data presented in Fig. 4b were obtained from the interface shear tests on Toyoura sand conducted by Stutz et al. [31], where a mild-steel surface with two different roughness levels was examined under a constant normal load of 78 kPa. The HvWE model predicts an earlier peak in the shear stress ratio at small displacements, which matches more with the experimental behavior for the intermediate surface roughness ($R_\textrm{max}$ = 20.5). For the rough surface conditions ($R_\textrm{max}$ = 46.0), the vector model provides a closer match to the experimental data, showing a prediction with higher initial stiffness and delayed peak. In addition, the simulations given by the vector model also gives a similar trend of the residual shear stress at critical state. Although for the proposed vector model, no additional parameters were introduced to account for the interface roughness, the simulations prove that the model still delivers satisfactory predictions across different roughness properties.

Figure 4c presents the response of a staged shear test in which the applied normal stress was intentionally changed during shearing [15]. The normal stress was initially maintained at 102 kPa and then increased to 274 kPa as the shear displacement reached 0.25 mm, creating a transition point in the stress –displacement curve. The interface was assumed to be fully rough. Both models successfully reproduce this transition point owing to the change in normal stress. However, the HvWE model overestimates the shear stress after the stress adjustment. As reaching the ultimate shear displacement, the test data indicates a strain-softening trend, characterized by a gradual reduction in shear stress near 2.5 mm. This post-peak behavior is accurately captured by the vector-based model, proving the enhanced predictive capability of the vector model as in-plane stress are considered.

An example of a CNS test implemented by Porcino et al. [28] is considered in Fig. 4d. In this test, the interface was sheared under a prescribed normal stiffness condition of *K* = 100 kPa, leading to a continuous increase in normal stress with shear displacement. Although the shear behavior of the HvWE model shows a similar behavior to the experimental results, it experiences a softening after the peak, which was not observed in the experiments. The vector model capture both the monotonic increase and subsequent stabilization of the stresses, whereas it shows less stiffness as loading starts. For the normal stress, neither model matches the ultimate experimental result. The HvWE model simulates a small contractive state followed by continued shearing to dilative behavior. Nevertheless, the vector model does not reproduce this transition in normal stress, instead yielding a steady dilative response consistent with the continuously increasing confinement under CNS conditions.

### Post-localization analysis of simple shear tests

To illustrate the capabilities of the strain-gradient extension of the proposed vector-based hypoplastic model, numerical simulations of simple shear tests are carried out. Simple shear tests are hereby adopted as a benchmark case for conducting the shear-band analysis enabled by the gradient-enhanced model. Under simple shear conditions, the kinematics of the granular material reduces to a single velocity component that depends only on one spatial coordinate. In this case, the analyses of the inherent length and shear-band formation can be conducted in a simplified framework: the general determinants involving the coefficients $\eta _{ij}$ and $b_i$ degenerate into single stiffness coefficients, and the norm of the strain-rate tensor $||\dot{\boldsymbol{\epsilon }}||$ reduces to the absolute value of the active strain-rate component.

Simple shear with a velocity field of $v_2(x_1) = a \sin (k x_1)$ reduces Eq. (20) with the condition $\dot{\sigma }_{12} = 0$ to

30 $$\begin{aligned} \eta _{22} \frac{dv_2}{dx_1} - \lambda ^2\eta _{22}\frac{d^3v_2}{d{x_1}^3} + \frac{1}{2} b_2 \left| \frac{dv_2}{dx_1} \right| = 0 \end{aligned}$$

Since $a/\left| a \right| = \pm 1$, the normalized wave length is obtained as

31 $$\begin{aligned} \frac{l}{\lambda } = 2\pi \left( \frac{1}{2} \left| \frac{\eta _{22}}{b_2} \right| - 1 \right) ^{-1/2} \end{aligned}$$

Here, $\lambda$ is the introduced length-scale parameter, while *l* represents the wavelength of the assumed localization mode. This solution exists if and only if

32 $$\begin{aligned} \frac{1}{2} \left| \frac{ \eta _{22}}{ b_2}\right| > 1 \end{aligned}$$

Therefore, for the velocity field $v_2(x_1)$, the hypoplastic stress increment in the shear component is given by

33 $$\begin{aligned} \dot{\sigma }_{12}=\eta _{22} \frac{\partial v_2}{\partial x_1} + \frac{1}{2} b_2 \left| \frac{\partial v_2}{\partial x_1} \right| \end{aligned}$$

**Fig. 5:**
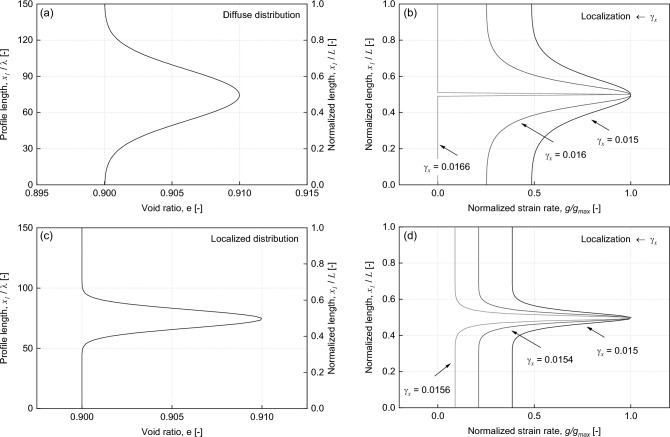
Shear-band analysis calculated by the non-gradient vector model in Eq. (10): **a**, **b** Diffuse distribution in an inhomogeneous sample and strain rate $g = \partial v_2/\partial x_1$ during different shear strains, **c**, **d** Localized distribution in an inhomogeneous sample and strain rate $g = \partial v_2/\partial x_1$ during different shear strains

In the numerical simulations of the extended model, simple shear is performed within a layer between $x_1$ = 0 and $x_1$ = *L* with prescribed velocities at boundaries:

34 $$\begin{aligned} v_2(0, t) = 0,\quad v_2(L, t) > 0 \end{aligned}$$

The equilibrium equation for the shear stress component $\dot{\boldsymbol{\sigma }}_{12} = 0$ in Eq. (30) becomes a fourth-order differential equation after time differentiation:

35 $$\begin{aligned} \frac{d}{dx_1}\left( \eta _{22} \frac{dv_2}{dx_1} - \lambda ^2\eta _{22}\frac{d^3v_2}{d{x_1}^3} + \frac{1}{2} b_2 \left| \frac{dv_2}{dx_1} \right| \right) = 0 \end{aligned}$$

Owing to the gradient term, two additional boundary conditions are required in addition to the displacement constraints:

36 $$\begin{aligned} \left. \frac{d^2 v_2}{d x_1^2} \right| _{(0, t)} = 0, \quad \left. \frac{d^2 v_2}{d x_1^2} \right| _{(L, t)} = 0 \end{aligned}$$

They ensures homogeneous deformation far away from the localized shear band. Physically, these conditions enforce that the velocity field outside the band remains linear, consistent with the original non-gradient formulation.

Two representative initial inhomogeneities in the void ratio (0.90–0.91) were considered, following the classical setups used in shear-band analysis by Osinov and Wu [26]. The diffuse distribution (Fig. 5a) describes a smoothly varying void ratio field, whereas the localized distribution (Fig. 5c) corresponds to a sharper and more concentrated perturbation near the middle of the material. Since the hypoplastic model is formulated in rate form, the stress integration is performed using a simple one-step Euler forward scheme [45]. Note that the gradient extension does not alter the fundamental rate structure of the model. The material parameter is listed in Table 3. The initial stress state of the simulation is taken as $\sigma _{n}$= 100 kPa, $\tau _{x}$ = $\tau _{y}$ = 0.

**Table 3:** Material parameters for simulating simple shear tests

Parameter	$E_i$ (MPa)	$\phi _c$	$\nu _i$	$e_d$	$e_{\Gamma }$	$\zeta$	$\xi$	$\alpha$
Value	13.0	30	0.28	0.55	0.95	0.102	0.107	0.3

The simulation results of the simple shear test using the non-gradient vector model (Eq. 10) are shown in Fig. 5. The evolution of shear localization is shown as the normalized strain rate, $g = \partial v_2 / \partial x_1$, plotted along the normalized profile length, $x_1 / L$. The results indicate that the initial inhomogeneity in the void ratio leads to a corresponding spatial variation in the initial strain rate field. As the in-plane shearing proceeding, the strain rate becomes to a concentration in the vicinity of the middle profile, where weak point with the largest void ratio is located. With shear strain reach to 0.014, the strain rate localized in a very thin area, only connected to the maximal void ratio. Note that the non-gradient vector model leads to a null solution beyond this stage of deformation.

**Fig. 6:**
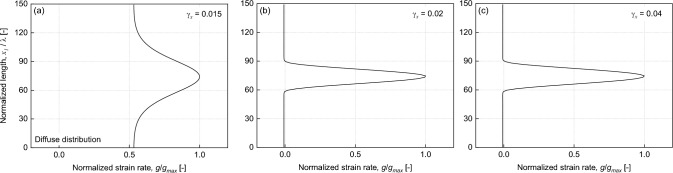
Normalized strain rate $g = \partial v_2/\partial x_1$ during different shear strains with a diffuse distribution in an inhomogeneous sample, calculated with the gradient model in Eq. (19)

**Fig. 7:**
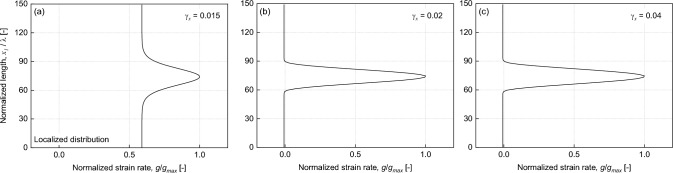
Normalized strain rate $g = \partial v_2/\partial x_1$ during different shear strains with a localized distribution in an inhomogeneous sample, calculated with the gradient model in Eq. (19)

The simulation results of the inhomogeneous samples with diffuse and localized distributions of the void ratio field calculated by the gradient model (Eq. 19) are shown in Figs. 6 and 7, respectively. Compared with the results from the non-gradient model (Fig. 5), the strain-gradient extension allows the vector model to reproduce a shear band of finite thickness in the post-localization stage and remain stable at larger shear strains. Furthermore, the results at $\gamma _{x} = 0.015$ indicate that the initial distributions of void ratio have negligible influence on the shear-band thickness after localization. This proves that the inherent length scale introduced by the gradient extension is governed by the material property, such as grain size, while it is essentially independent of the initial density distribution.

### Influence of contact surface on localization

In soil-structure interface problems, the shear-band thickness may be influenced by the characteristics of the contact surface, such as interface roughness. In this study, the influence of the contact surface on strain localization of granular material is further investigated by giving a coefficient $\chi$ into the strain-gradient boundary condition of the gradient-enhanced model. The objective is to investigate how contact surface affects the onset of localization and the resulting shear-band thickness in the post-localization regime. In this way, the interface behavior can be represented without introducing any additional material parameters, preserving the simplicity of the vector hypoplastic model. Under simple shear conditions, the boundary condition in Eq. (36) becomes:

37 $$\begin{aligned} \left. \frac{d^2 v_2}{d x_1^2} \right| _{(0, t)} = 0, \quad \left. \frac{d^2 v_2}{d x_1^2} \right| _{(L, t)} = - \chi \end{aligned}$$

where the coefficient $\chi$ imposes a curvature in the strain-gradient field at the boundary, represented by a negative strain gradient at the interface with the profile length of $x = L$. It controls the curvature of the shear strain profile and can be related to the surface geometry. Compare to the free boundary condition in Eq. (36), the coefficient $\chi$ in Eq. (37) acts as a localization driver, leading an earlier onset of localization regime and thinner shear band than that observed under free boundary conditions.

**Fig. 8:**
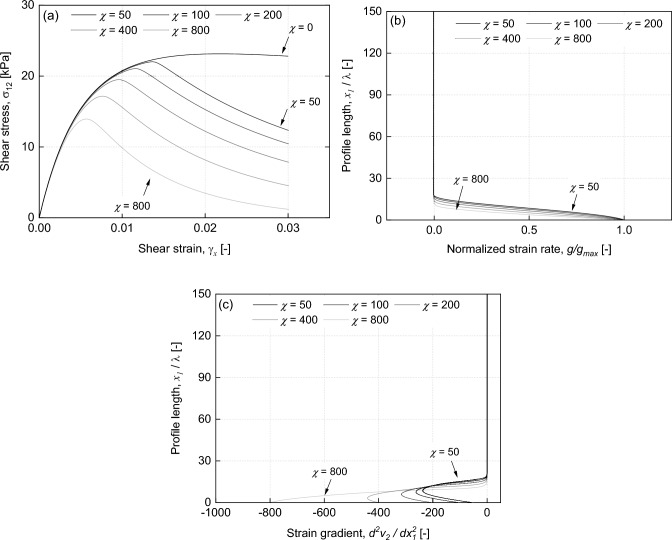
Shear-band analysis of interface behavior by the vector-based hypoplastic model in Eq. (10). **a** Shear stress ($\tau _{x}$) vs. Shear strain ($\gamma _x$). **b** Strain rate $g = \partial v_2/\partial x_1$ of the homogeneous samples with boundary conditions given in Eq. (37). **c** Strain gradient ${d^2 v_2}/{d x_1^2}$ of the homogeneous samples with boundary conditions given in Eq. (37)

Numerical simulations on interface behavior are conducted with strain-gradient extensions (Eq. 19) on the proposed vector model in Eq. (10). Since localization is now implemented at the interface boundary, the inhomogeneous distribution of the void ratio field was no longer needed. Instead, a homogeneous sample with an initial void ratio of $e_0 = 0.90$ was used. Figure 8 shows the simulation results for the interface shear-band analysis. The initial stress state was adopted as $\sigma _{n}$ = 100 kPa.

The results show that the coefficient $\chi$ effectively characterizes the post-localization behavior during interface shear in homogeneous granular materials. As shown in Fig. 8a, the increasing $\chi$ leads to the earlier onset of strain localization, accompanied by the more pronounced strain-softening behavior with the reduced shear stress. Additionally, the imposed curvature arisen by $\chi$ at the interface boundary changes the thickness of the interface shear band. As shown in Fig. 8b, localization occurs at the lower boundary surface, while the material outside the shear-band exhibits a nearly rigid deformation of strain rate close to zero. As $\chi$ increases, the strain rate distribution becomes more concentrated, indicating highly localized deformation regulated by a smoother interface. The corresponding strain gradient profiles are presented in Fig. 8c. For a small $\chi$, the curvature of the strain gradient changes sharply near the boundary, indicating the presence of rough surface asperities.

The coefficient $\chi$ provides a phenomenological measure of the boundary influence in the gradient-enhanced model. By varying $\chi$, different levels of boundary constraint can be represented within the constitutive framework, without introducing additional material parameters. Consequently, the onset of localization and the resulting shear-band thickness of granular material can be investigated under different contact surface boundary conditions.

## Conclusions

A vector-based hypoplastic model is presented to describe the interface behavior of granular materials. By introducing the unit normal vector of the interface, the tensorial hypoplastic model is reduced to vector quantities representing the in-plane projections of stress and strain rate. The proposed vector-based model remains invariant under coordinate rotation and allows the interface to be oriented arbitrarily, since all quantities transform according to standard geometric rules.

A series of numerical tests is carried out to validate the proposed model under different interface boundary conditions. The vector-based hypoplastic model effectively captures the salient behavior of granular soils with different initial densities. The comparison with experimental data shows that the vector-based model well reproduces the post-peak response given by dense soil and the hardening behavior governed by rough surface during interface tests. To conduct the shear-band analysis in post-localization regime, a strain-gradient term is incorporated into the vector-based model with a length scale parameter. The simulation results show that the gradient-enhanced vector model predicts a shear band with finite thickness in the post-localization regime, which is independent of the initial inhomogeneity. Furthermore, earlier onset of localization and reduced shear-band thickness in granular soil can be achieved by modifying the strain-gradient boundary condition associated with the contact surface.

## Data Availability

The data that support the findings of this study are available from the corresponding author upon reasonable request.

## Vector-based hypoplastic model

The vector-based hypoplastic model for interface behavior is given as:

A.1 $$\begin{aligned} \dot{\boldsymbol{\sigma }}_{{\textbf {v}}}= f_s\Big [\sigma _n{\dot{\boldsymbol{\epsilon }}}_{{\textbf {v}}} + f_v\dot{\epsilon }_{n}\boldsymbol{\sigma }_{{\textbf {v}}} + a^2\frac{\boldsymbol{\sigma }_{{\textbf {v}}}\cdot \dot{\boldsymbol{\epsilon }}_{{\textbf {v}}}}{\sigma _n}\boldsymbol{\sigma }_{{\textbf {v}}} + f_da(\boldsymbol{\sigma }_{{\textbf {v}}}+\boldsymbol{\sigma }_{{\textbf {v}}}^*)\left\| \dot{\boldsymbol{\epsilon }}_{{\textbf {v}}}\right\| \Big ] \end{aligned}$$

where $\sigma _n$ and $\dot{\epsilon }_n$ representing the stress and strain rate component perpendicular to the interface, obtained by:

A.2 $$\begin{aligned} \sigma _n = \boldsymbol{\sigma }_{{\textbf {v}}} \cdot \boldsymbol{n}, \quad \dot{\epsilon }_{n} = \dot{\boldsymbol{\epsilon }}_{{\textbf {v}}} \cdot \boldsymbol{n} \end{aligned}$$

where $\boldsymbol{n} = [1,0,0]^\textit{T}$ is the unit normal vector. It can rotate according to interface angle, e.g., $\boldsymbol{n} = [0,1,0]^\textit{T}$ or $\boldsymbol{n} = [0,0,1]^\textit{T}$. The deviatoric stress is derived by:

A.3 $$\begin{aligned} \boldsymbol{\sigma }_{{\textbf {v}}}^* = \boldsymbol{\sigma }_{{\textbf {v}}} - (\boldsymbol{\sigma }_{{\textbf {v}}} \cdot \boldsymbol{n})\boldsymbol{n} \end{aligned}$$

Multipliers $f_s$ and $f_v$ accounts for the stiffness and volumetric response of the materials, respectively:

A.4 $$\begin{aligned} f_s = -\frac{E_i}{(1+\nu _i)\sigma _c},\; f_v = \frac{3\nu _i+a\sqrt{1+2\nu _i^2}}{2(1-2\nu _i)}- \frac{5a^2}{6} \end{aligned}$$

in which $E_i$ and $\nu _i$ are material parameters. $\sigma _c =100$ kPa is used for material calibration.

The density factor $f_d$ is given as follows:

A.5 $$\begin{aligned} f_d=\left( \frac{e-e_\text {d}}{e_\text {c}-e_\text {d}}\right) ^\alpha \end{aligned}$$

where $\alpha$ is a constant that controls the degree of strain softening; *e* and $e_{d}$ are the current and minimum void ratios, $e_{c}$ is the critical state void ratio:

A.6 $$\begin{aligned} e_{c} = e_{\Gamma } \exp \left[ -\zeta \left( \frac{p}{p_{a}} \right) ^{\xi } \right] . \end{aligned}$$

where $e_\Gamma$, $\zeta$, and $\xi$ are material parameters for characterizing the critical state of granular materials. $p^\prime$ is the effective mean stress and $p_\text {a}$ = −101.325 kPa is the atmospheric pressure for normalization.

The factor *a* regulates the strength of the material through:

A.7 $$\begin{aligned} a = \frac{\sqrt{3}(2\sqrt{2} - \sin \phi _c)}{4\sqrt{2} \sin \phi _c} \end{aligned}$$

where $\phi _c$ is the critical state friction angle.

There are seven parameters for the proposed model: $E_i$, $\nu _i$, $\phi _c$, $\alpha$, $e_\Gamma$, $\zeta$, and $\xi$. $E_i$ (initial elastic modulus), $\nu _i$ (initial Poisson’s ratio), and $\phi _c$ (critical state friction angle) can be obtained from a single triaxial compression test with confining pressure of 100 kPa. $\alpha$ can be determined by trial and error based on the density effect on strain softening or hardening. The critical state parameters $e_{\Gamma }$, $\zeta$, and $\xi$ can be measured from an $e-\text {log}(p')$ plot of critical states based on three triaxial compression tests.

## Derivation of the inherent-length analysis

For the inherent-length analysis, the governing ordinary differential equations are written in algebraic form by assuming a sinusoidal velocity field. This leads to a homogeneous linear system for the normalized amplitude $\alpha _i$, written as:

B.1 $$\begin{aligned} \alpha _i = \frac{(1 - \lambda ^2 \xi ^2) \Lambda _i}{\Lambda _0}, \quad i = 1, 2, 3 \end{aligned}$$

where the determinants $\Lambda _0$, $\Lambda _1$, $\Lambda _2$, and $\Lambda _3$ depend on the coefficients $\eta _{ij}$ and $b_i$, given as:

B.2 $$\begin{aligned} \begin{aligned} \Lambda _0&= \det \begin{pmatrix} \eta _{11} &  \eta _{12} &  \eta _{13} \\ \eta _{21} &  \eta _{22} &  \eta _{23} \\ \eta _{31} &  \eta _{32} &  \eta _{33} \end{pmatrix}, \\ \Lambda _1&= \det \begin{pmatrix} -b_1 &  \eta _{12} &  \eta _{13} \\ -b_2 &  \eta _{22} &  \eta _{23} \\ -b_3 &  \eta _{32} &  \eta _{33} \end{pmatrix}, \\ \Lambda _2&= \det \begin{pmatrix} \eta _{11} &  -b_1 &  \eta _{13} \\ \eta _{21} &  -b_2 &  \eta _{23} \\ \eta _{31} &  -b_3 &  \eta _{33} \end{pmatrix}, \\ \Lambda _3&= \det \begin{pmatrix} \eta _{11} &  \eta _{12} &  -b_1 \\ \eta _{21} &  \eta _{22} &  -b_2 \\ \eta _{31} &  \eta _{32} &  -b_3 \end{pmatrix}. \end{aligned} \end{aligned}$$

This derivation leads directly to the characteristic equation presented in Eq. (22).
